# Effect of age at initiation of antiretroviral therapy on treatment outcomes; A retrospective cohort study at a large HIV clinic in southwestern Uganda

**DOI:** 10.1371/journal.pone.0201898

**Published:** 2018-08-15

**Authors:** Peter Ssebutinde, Imelda T. Kyamwanga, Eleanor Turyakira, Stephen Asiimwe, Francis Bajunirwe

**Affiliations:** 1 Mbarara District Directorate of Health Services, Mbarara, Uganda; 2 Department of Community Health, Mbarara University of Science and Technology, Mbarara, Uganda; 3 Kabwohe Clinical Research center, Bushenyi, Uganda; Universita degli Studi di Roma Tor Vergata, ITALY

## Abstract

**Background:**

The prevalence of HIV infection among older persons is increasing yet older age at initiation of antiretroviral therapy (ART) may be associated with poorer treatment outcomes including mortality. However, majority of these studies have been done in the western world and there is limited data in resource limited settings. Our study used routinely collected health facility data to assess trends in age at initiation of ART, the effect of age at ART initiation on mortality and immunological response at a large urban hospital in south western Uganda.

**Methods:**

We conducted a retrospective records review of patients attending the HIV clinic at Mbarara Regional Referral Hospital in western Uganda. We retrieved records for 8,533 patients who started ART between January 2006 and December 2012. Their data had been collected and stored as part of the larger International Epidemiological Database for the Evaluation of AIDS (IeDEA). Age was stratified into three categories namely; 18–34 (young adults), 35–49 (mid-age) and 50 years or older (older adults). Survival analysis procedures with Kaplan-Meier’s plots were used to calculate the survival probability with mortality as the endpoint and Poisson regression analysis used to determine the adjusted relative risks (RR) of mortality.

**Results:**

The proportion of young adults and patients at WHO stage I initiating ART increased steadily over the 7-year period. Older age at ART initiation (> = 50 years) was associated with a higher risk of mortality with adjusted relative risk (RR) at 1.63, (95% CI 1.26–2.11) compared to younger age. Male gender, WHO stages III and IV, lower CD4 count and lower body mass index were also all independently and significantly associated with higher risk for mortality. Older adults also had a poorer immunological response RR = 1.79 (95% CI 0.89–3.58) but was not statistically significant.

**Conclusions:**

Following ART initiation, older adults compared to the young, have a higher risk of mortality. This age group should be targeted first for ‘screen and treat’ approach. Optimization of ART treatment regimens for this age group is also required to reduce mortality and improve immunological response.

## Introduction

The prevalence of HIV infection among persons aged 50 years and over is steadily rising and globally in 2013, an estimated 4.2 million people in this age category were living with HIV/AIDS [1], with the number doubling since 1995. HIV infected patients aged 50 years and over are considered ‘elderly’. Almost 60% of them live in sub Saharan Africa. In Uganda, HIV infection among persons aged 50 years and above is increasing and estimated to be about 11.3%, a figure much higher than in the general population where prevalence according to the UNAIDS report [2] of 2013, is at 7.3%.

Mortality of HIV infected persons has significantly reduced in the era of antiretroviral therapy [3, 4]. Antiretroviral therapy (ART) is associated with increased survival among HIV infected patients in both high income [5, 6] and low income settings [7, 8]. The infected persons, regardless of income status of country of residence, can now expect to live to their normal life expectancy especially if care and treatment are initiated in a timely fashion. However, age at initiation of treatment may modify the effect of antiretroviral therapy on the outcomes. Studies to investigate the relationship between age at initiation of ART and treatment outcomes have shown mixed results. Some studies show older age is associated with slower or poorer immunological response and a higher mortality risk compared to the younger ones after ART initiation [9–11], yet others have shown no differences in treatment outcomes among younger and older patients aged more than 50 years [12]. In this study, differences emerged only when analyses considered comparisons with patients in the extreme age groups of above 60 years. Older patients also represent an important subgroup because they are at risk for a delayed HIV diagnosis compared to younger patients [13, 14]. The delay in seeking an HIV test may be due to structural barriers such as stigma [15, 16] or limited access to HIV testing services [17, 18].

Majority of the studies on impact of age on response to ART have been conducted in the developed countries, and to-date fewer studies in the resource limited settings. The few studies in Africa [19, 20] suggest the proportionate mortality rate due to HIV/AIDS among older adults may be higher in low income countries compared to the richer countries. The higher frequency of HIV comorbidities in sub Saharan Africa including Tuberculosis (TB) and other opportunistic infections and parasites may explain the difference. However, the duration of patient follow up in most of these studies was shorter. Therefore, more data from treated HIV cohorts with long term follow up in resource limited settings are necessary to understand the differences in mortality.

Understanding the impact of age at ART initiation on the immunological response and mortality will inform clinical service and assist in making policy and recommendations regarding treatment guidelines for older patients (≥ 50years). Older patients might require earlier initiation of treatment if advanced age is associated with poorer response and survival following treatment. The current WHO (2016) treatment guidelines [21] recommend that treatment should be initiated regardless of WHO stage of disease or CD4 count shifting towards a test and treat approach. Given the resource constraint for most countries with a high burden of HIV, it will be difficult to achieve the test and treat for all HIV positives. This data may provide additional evidence to support recommendations for priority in prevention and treatment of older HIV infected patients with ART in the implementation of test and treat.

The prevalence of HIV among older persons is increasing and presenting potential temporal trends in the age distribution of patients at the HIV clinics in sub Saharan Africa. Understanding the temporal trends of age at initiation of ART over an extended period may be used to mirror disease burden or incidence of HIV in the general population. In a mature and generalized epidemic like that in Uganda, it is likely there have been temporal trends in age distribution at initiation of ART. It may be argued that age distribution at initiation of ART may reflect incidence of HIV in the general population, especially if there are no barriers in access to HIV treatment services by certain age groups. The data may shed light on the age groups that are at highest risk for HIV incidence. We used routinely collected data at a large HIV clinic to determine temporal trends in age at ART initiation among these patients, assess the effect of age at ART initiation on mortality and immunological response after initiation of ART.

## Methods

### Study design

We conducted a retrospective cohort study at a large urban HIV clinic in the south western Ugandan town of Mbarara. The clinic is based within Mbarara Regional Referral Hospital, a teaching hospital for Mbarara University medical school. The clinic was established in 1998 and by December 2012, there were 22,643 cumulative and 9,329 active entries of patients receiving ART.

The retrospective cohort was constructed using routinely collected data at the clinic. The data for analysis was extracted from the International Epidemiological Database to evaluate AIDS (IeDEA) of Mbarara Regional Referral Hospital. The hospital joined the IeDEA network in January 2006, which now exists at 3 other sites in Uganda. Our analysis only uses data from Mbarara Hospital. All patients enrolled in care at the regional referral hospital are entered into this database. Patients initiated on ART before January 2006 were not entered in IeDEA. Therefore, we considered patients initiated on ART from January 1, 2006, when the IeDEA database started to December 31, 2012. We extracted several variables including socio-demographic factors, clinical and laboratory data like CD4 counts, viral load, BMI at initiation of ART, WHO clinical staging, ART start date and ART regimen. Patients in the IeDEA database were eligible for analysis if they were aged ≥18 years old, and had initiated ART on or before December 31, 2012.

Laboratory biomarkers such as CD4 counts, Complete Blood Count and Hemoglobin for patients at initiation of ART were collated with the patients’ routine follow-up visits information.

### Data analysis

The data were cleaned to ensure consistency in the coding of variables and for missing data. Patients with missing age were excluded from the analysis. Patients who were missing other variables were included in the analysis but were automatically excluded in any analysis where the missing variable was involved. No data imputation was done. Ages for patients initiated on ART were stratified in three categories namely; 18 to 34 years (young adults), 35 to 49 years (mid-age) and ≥50 years (older adults). The age categories were considered as the main exposure variable. CD4 change as a predictor was created by obtaining the difference between the most recent and CD4 cell count at ART initiation. The variable CD4 change was broken into 4 categories: CD4 decline, CD4 increase of <100, CD4 increase of 100–350 and CD4 increase >350. Baseline characteristics across the age strata were compared using chi square and those that presented differences were included in the multivariable analysis.

The primary endpoint for this analysis was mortality after initiation of ART and the secondary endpoint was immunologic response. We used Survival analysis to determine the effect of age on mortality using the Kaplan Meier plot and the Log rank test. We report the median survival and the inter quartile range (IQR). Poisson regression analysis was used to determine the rate ratios for effect of age on mortality and immunological response. Immunological response was a secondary outcome and was classified by dichotomizing the most recent CD4 into either CD4 count below 350 or ≥350. The patients with CD4 cell count less than 350 were classified as having a poor immunological outcome. A multi variable analysis was done using Poisson regression modeling with the predictor variables that were considered confounders entered into the model simultaneously, using a cut off of p<0.1 in the bivariate analysis as consideration for inclusion in the multiple regression model. All analyses were completed using STATA version 11 (College Station, TX).

### Ethical considerations

The study protocols were approved by the Department of Community Health, the HIV clinic data sharing committee, the Faculty of Medicine Research ethics committee and the Mbarara University of Science and Technology Research Ethics Committee. All the datasets extracted from the IeDEA database were kept under password lock. Data were de-identified and codes instead of names were used and all the information was kept confidential.

## Results

### Baseline characteristics and temporal trends

**Fig 1:**
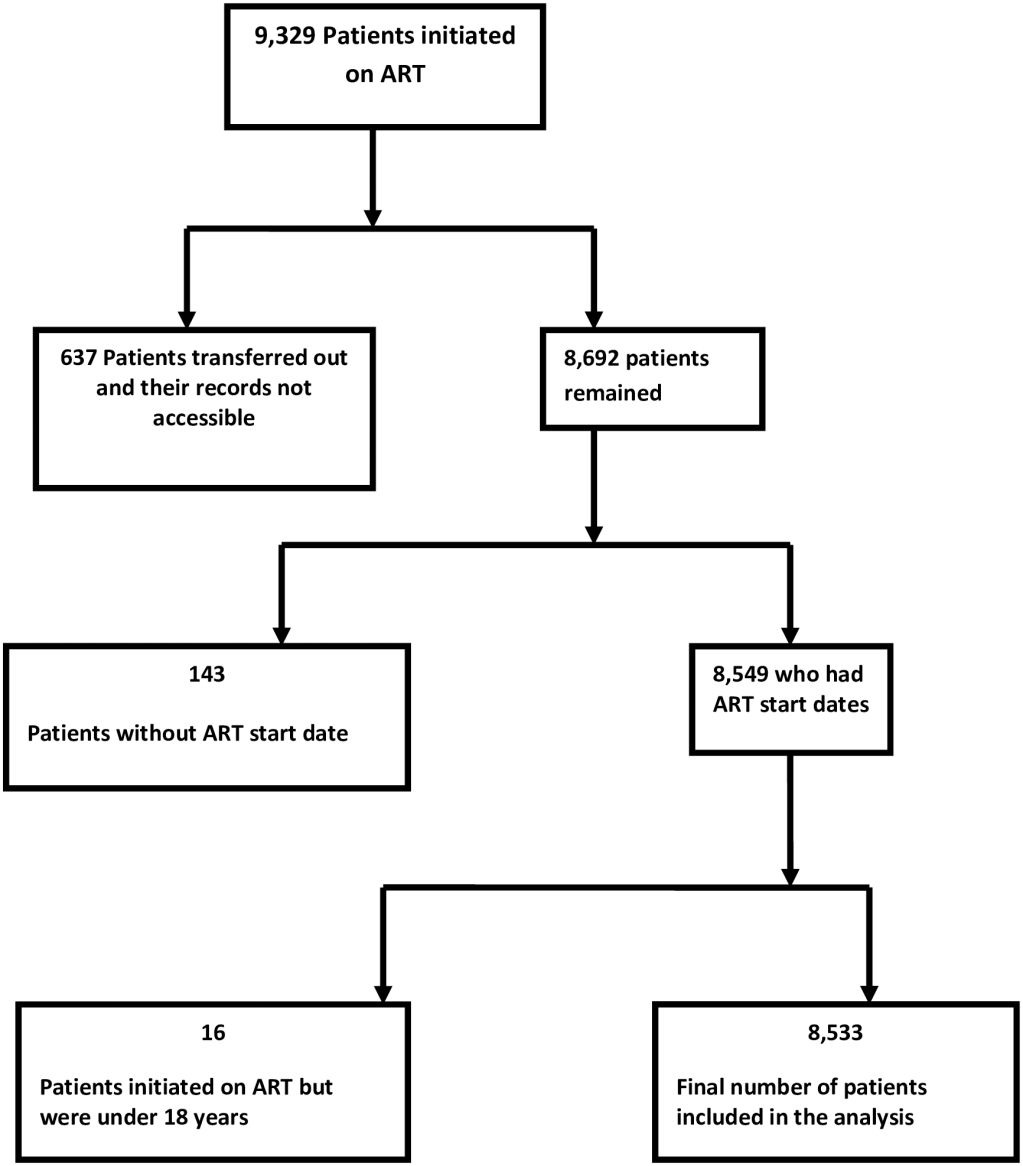
Summary profile of patients enrolled in HIV treatment at Mbarara Regional Referral Hospital, Uganda, 2006–2012.

A total of 8,364 patients, who initiated ART between January 1, 2006 and December 31, 2012 were eligible and included in the analysis. The summary of the cohort is represented in Fig 1.

Of these, 5,308 (62.2%) were females. The cohort contributed a total of 66,644 person years (PYs) of observation with a median follow up time of 1.5 years (IQR 0.5, 3.9). The median age was 32 years with IQR 27, 39) and the distribution of the age strata were 4,686 (56.0%) young adults (18–34 years), 3,121 (37.3%) middle aged (35–49 years) and 557 (6.7%) patients with age ≥ 50 years. The younger patients were more likely to be female while the older patients were more likely to be male p = 0.0001. The distribution of the baseline characteristics among the three age strata is shown in Table 1.

**Table 1:** Baseline demographic characteristics among three age categories for patients initiated on ART at large HIV clinic (1^st^ January 2006 to 31^st^ December 2012).

Age Categories n (%)					
Characteristics		Young Adults (18–34years)	Middle aged (35–49 years)	Older patients (50years or over)	p value
**Gender (n = 6,556)**	Females	2, 739 (73.6)	1,259 (52.6)	204 (46.2)	
	Males	982 (26.4)	1,134 (47.4)	238 (53.8)	0.0001
**WHO stage (n = 6,284)**	I	2,095 (58.9)	1,328 (57.7)	243 (57.5)	
	II	724 (20.3)	465 (20.2)	185 (20.1)	
	III	638 (17.9)	439 (19.1)	82 (19.4)	0.0001
	IV	102 (2.9)	70 (3.0)	13(3.1)	
**CD4 Count (n = 6,556)**	CD4 <100	915 (24.6)	647 (27.0)	114 (25.8)	0.0001
	CD4 (101–250)	1,518 (40.8)	1,120 (46.8)	195 (44.1)	
	CD4 (251–350)	815(21.9)	496 (20.7)	106 (24.0)	
	CD4 > 350	473 (12.7)	130 (5.4)	27 (6.1)	
**Duration on ART (n = 6,555)**	< 6months	871 (23.40)	502 (21.0)	100 (22.6)	
	6–12 months	439 (11.8)	275 (11.5)	60 (13.6)	0.0001
	13–36 months	1,394 (37.5)	816 (34.1)	138 (31.2)	
	Above 36 months	1,016 (27.3)	800 (33.4)	144 (32.6)	
**Body Mass Index (n = 6,035)**	> = 30	103 (3.0)	63 (2.9)	15 (3.8)	
	25.0–29.9	112 (2.8)	66 (2.5)	13 (2.7)	
	18.5–24.9	2,552 (74.1)	1,459 (66.6)	244 (61.0)	0.0001
	<18.5	790 (22.9)	668 (30.5)	141 (35.3)	
**Education (n = 5,244)**	None	121 (4.0)	148 (7.9)	31 (10.2)	
	Primary	1,875 (61.4)	1,175 (62.3)	197 (65.2)	0.0001
	Secondary	816 (26.7)	381 (20.2)	44 (14.5)	
	Tertiary	244 (8.0)	182 (9.7)	30 (9.9)	
**Occupation (n = 5,745)**	Unemployed	684 (21.1)	337 (16.8)	59 (15.4)	
	Business	708 (21.8)	314 (15.6)	42 (10.9)	
	Farmer	978 (30.1)	840 (41.8)	207 (54.1)	<0.001
	Civil service	100 (3.1)	140 (6.9)	20 (5.2)	
	Others	779 (23.9)	381 (18.9)	55 (14.4)	
**Partner HIV status (N = 6,103**	Negative	189 (5.6)	179 (7.7)	38 (10.2)	
	Positive	1,519 (44.8)	1,049 (44.8)	129 (4.8)	0.381
	Unknown	943 (27.8)	670 (28.6)	96 (34.5)	
	No partner	738 (21.8)	442 (18.9)	101 (27.1)	

WHO stage, CD4 count at enrollment, body mass index and educational level were significantly different in the three age strata. Occupation and partner HIV status were not different in the three age strata.

**Fig 2:**
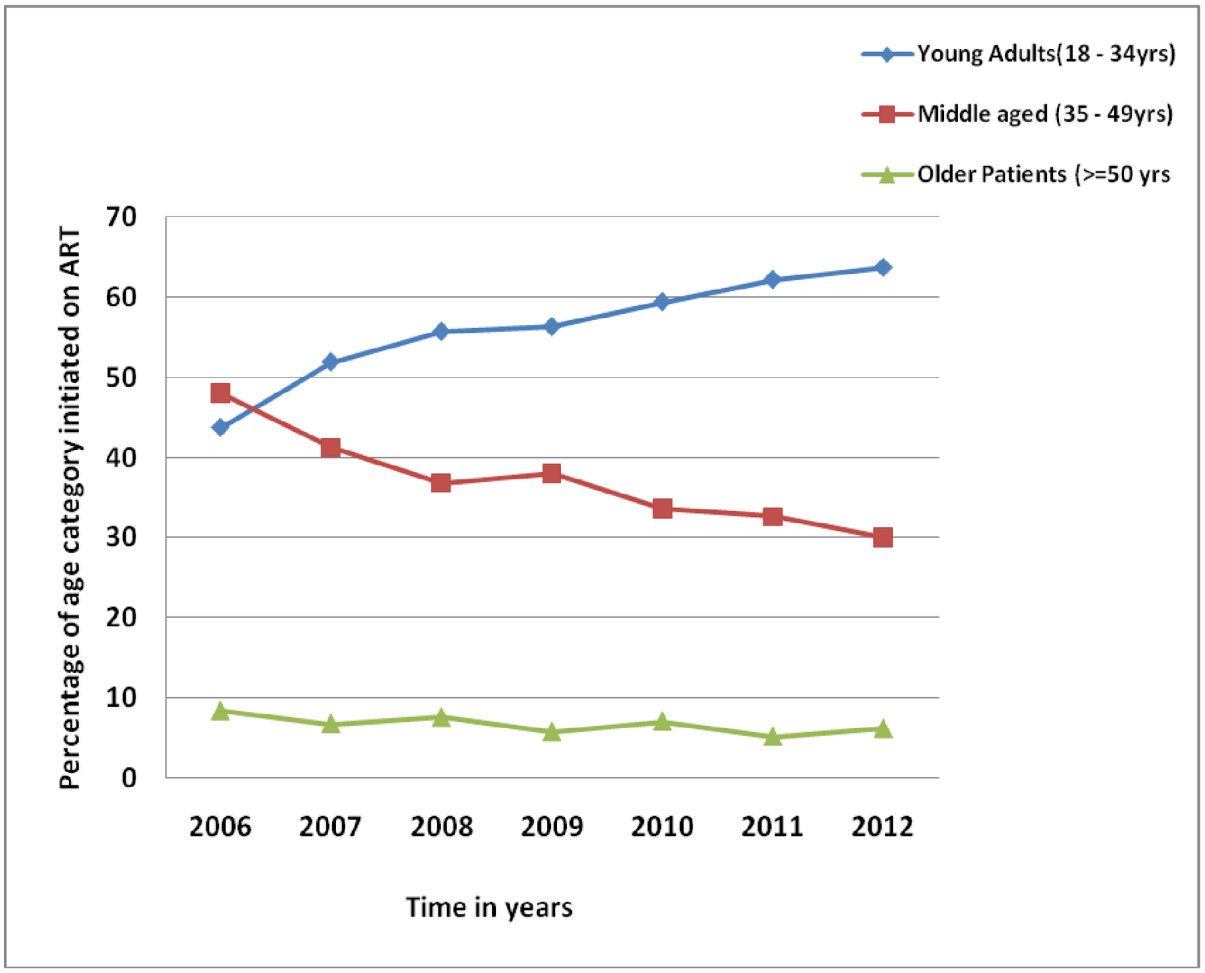
Trends in age of patients initiated on ART at Mbarara Regional Referral Hospital, Uganda, 2006–2012.

The proportion of young adults initiating ART has consistently increased from 42% in 2006 to 62% in 2012. The proportions for the other age groups have either remained stable or decreased (Fig 2).

### Kaplan Meier survival analysis

**Fig 3:**
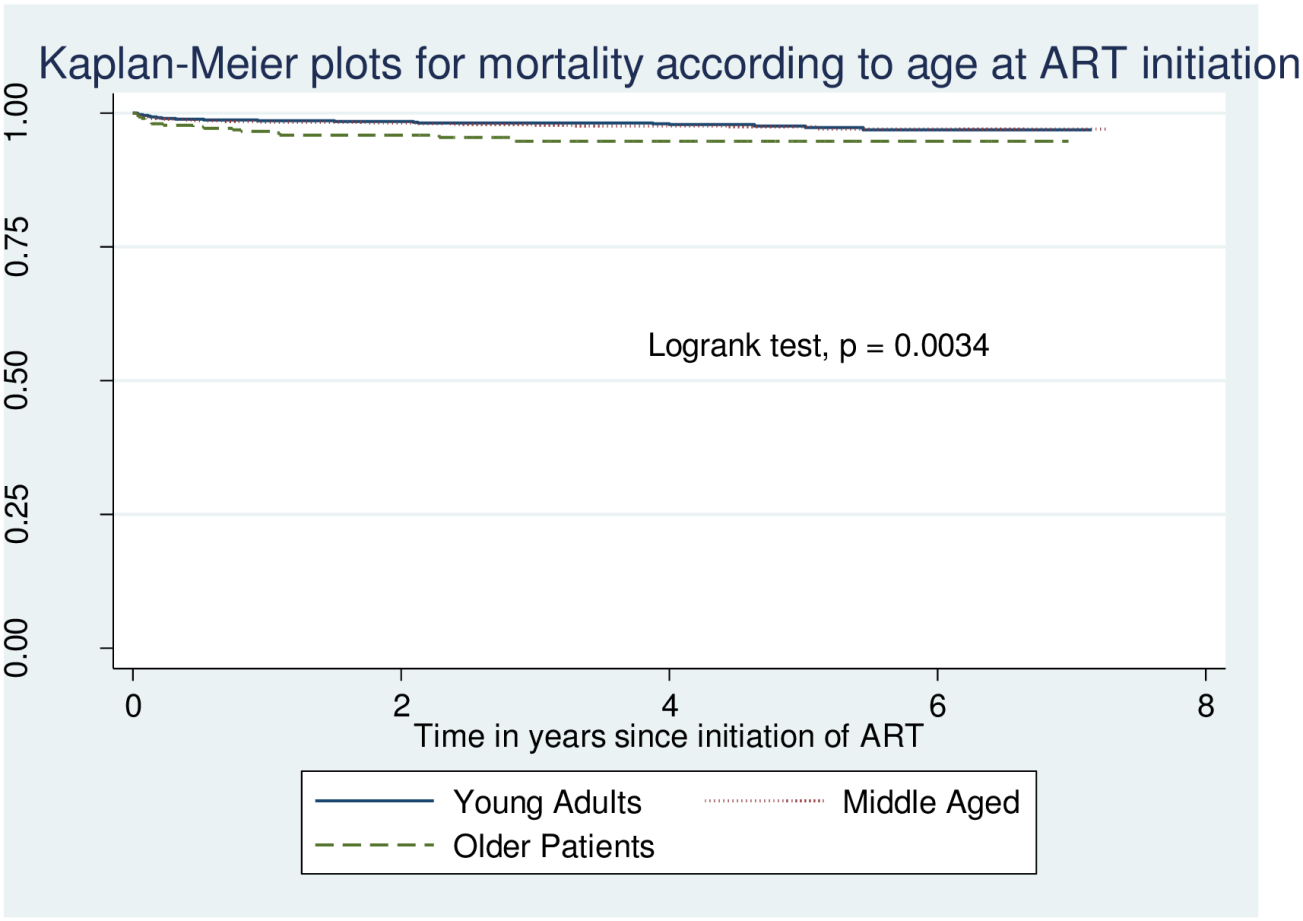
Survival by age at initiation of ART among patients at Mbarara Regional Referral Hospital, Uganda, 2006–2012.

Overall, 180 (2.1%) patients died. The Kaplan-Meier plot is shown in Fig 3 and the bivariate analysis indicates the older patients (≥50 years) had a significantly lower probability of survival compared to the young (Log rank test p = 0.0034). However, the curves for the middle aged and young adults appear superimposed over each other suggesting a lack of difference in mortality and survival in these two age groups.

Table 2 shows the proportion of young adults initiating ART steadily increased from 43.7% in 2006 to 63.7% in 2012 (p value for chi square test for trend = 0.002). Proportion of patients initiating ART with CD4 count over 350 doubled from 6.1% to 13.9% and patients in WHO stage IV decreased from 11.2% to 5.3% over the same period. Gender distribution remained stable with predominantly women at the clinic.

**Table 2:** Table showing trends in characteristics of patients at initiation on ART between January 2006 and December 2012, Mbarara Regional Referral Hospital, Uganda.

Variable	Categories	Year of ART initiation n (%)						
		2006	2007	2008	2009	2010	2011	2012
**Age category (n = 8,364)**	18–34 years	503 (43.7)	689 (51.9)	740 (55.7)	622 (56.3)	732 (59.4)	690 (62.2)	710 (63.7)
	35–49 years	552 (48.0)	550 (41.2)	488 (36.8)	420 (38.0)	414 (33.6)	362 (32.6)	335 (30.0)
	> = 50 years)	95 (8.3)	89 (6.7)	100 (7.5)	63 (5.7)	86 (7.0)	56 (5.1)	68 (6.1)
**Gender (n = 8,533)**	Females	721 (62.3)	862 (63.2)	856 (62.8)	711 (62.7)	785 (62.2)	689 (61.5)	687 (60.6)
	Males	436 (37.7)	501 (36.8)	509 (37.3)	422 (37.3)	477 (37.8)	431 (38.5)	446 (39.4)
**CD4 cell count at ART initiation/μml, (n = 6,560)**	<100	189 (25.0)	203 (20.8)	350 (30.2)	254 (27.0)	254 (25.7)	222 (25.2)	196 (23.7)
	101–250	395 (49.9)	510 (52.4)	521 (45.0)	424 (45.1)	410 (41.4)	284 (32.2)	291 (35.2)
	251–350	151 (19.1)	205 (21.1)	221 (19.1)	202 (21.5)	191 (19.3)	224 (25.4)	224 (27.1)
	> 350	48 (6.1)	55 (5.7)	66 (5.7)	60 (6.4)	135 (13.6)	151 (17.1)	115 (13.9)
**WHO stage at ART initiation (n = 7,665)**	I	198 (17.9)	260 (20.4)	368 (30.9)	418 (41.8)	521 (45.5)	566 (57.0)	584 (61.1)
	II	313 (28.3)	429 (33.7)	397 (33.4)	322 (32.2)	337 (29.4)	280 (28.2)	245 (25.6)
	III	473 (42.7)	460 (36.1)	330 (27.8)	202 (20.2)	181 (15.8)	89 (9.0)	76 (8.0)
	IV	124 (11.2)	125 (9.8)	94 (7.9)	58 (5.8)	106 (9.3)	58 (5.8)	51 (5.3)
**Body Mass index (N = 7,075)**	Under weight	261 (29.0)	366 (31.2)	315 (29.4)	287 (30.3)	326 (29.2)	200 (21.2)	200 (21.7)
	Normal	611 (67.8)	758 (64.7)	721 (67.3)	634 (66.9)	744 (66.7)	701 (74.2)	683 (74.0)
	Over weight	23 (2.6)	423.6)	28 (2.6)	23 (2.4)	29 (2.6)	41 (4.3)	34 (3.7)
	Obese	6 (0.7)	6 (0.5)	7 (0.7)	4 (0.4)	161.4)	3 (0.3)	6 (0.7)

Table 3 shows the crude and adjusted risk ratios for the predictors of mortality. The adjusted model shows that older age was associated with a 63% increased risk of mortality (adjusted RR = 1.63, 95% C.I 1.26–2.11) compared to the young adults while as middle aged had a 17% reduced risk for mortality compared to the young. Men had a 45% higher risk for mortality compared to the females (adjusted RR = 1.45, 95% CI 1.23, 1.72). Mortality risk was higher among patients initiated on ART at more advanced disease stage of 2, 3 or 4 compared to those initiated on ART at disease stage 1. Similarly, there was a high risk for mortality in the lowest CD4 category (less than 100) compared to the highest category of more than 350 cells, with a dose response relationship. The multivariable regression analysis shows that after adjusting for gender, CD4 count, education and duration on ART, age at initiation of ART remained an important predictor of mortality and older patients had a poorer prognosis compared to the young age group.

**Table 3:** Table showing crude and adjusted risk ratios for predictors of mortality using Poisson regression analysis.

Variable	Categories	Crude Risk Ratios (95% CI)	p value	Adjusted^+^ Risk Ratios (95% CI)	p value
**Age Categories**	Young adults (18–34 years)	1.0		1.0	
	Middle aged (35–49 years)	1.11 (0.79–1.35)	0.029	0.83* (0.69–0.98)	0.037
	Older (> = 50 years)	2.03* (1.25–3.30)		1.63* (1.26–2.11)	
**Gender**	Female	1.0		1.0	
	Male	2.33* (1.71–3.17)	<0.001	1.45* (1.23–1.72)	0.008
**WHO Stage**	I	1.0		1.0	
	II	4.50* (2.46–8.12)		2.89* (2.17–3.85)	
	III	22.20* (13.56–36.21)	<0.001	9.17* (7.22–11.65)	<0.001
	IV	96.64* (51.81–162.09)		18.14* (13.63–24.15)	
	
**CD4 count**	> 350 cells/ml	1.0		1.0	
	251–350 cells/ml	2.20 (0.5–9.67)		4.32* (1.89–8.94)	
	100–250 cells/ml	3.23 (0.79–13.20)	<0.001	5.66* (2.66–12.05)	0.005
	<100 cells/ml	7.79* (1.92–31.67)		7.32* (4.14–18.79)	
**Level of Education**	None	1		1	
	Primary	0.88 (0.49–1.54)		0.51* (0.39–0.67)	
	Secondary	0.75 (0.38–1.45)	0.052	0.51* (0.37–0.68)	0.09
	Tertiary	1.35 (0.67–2.70)		1.16 (0.83–1.62)	
**Body Mass Index**	> = 30	1		1	
	25.0–29.9	0.28* (0.12–0.66)	<0.001	0.33* (0.25–0.60)	<0.001
	18.5–24.9	0.42 (0.71–1.34)		0.35 (0.69–1.13)	
	<18.5	1.05 (0.46–2.43)		0.62 (0.29–1.23)	

* Significant at 0.05 level.

^+^The variables adjusted for include gender, CD4 count, BMI, level of education

In Table 4, we present the results for the analysis to determine the effect of age on CD4 response. The older age group had an increased risk of 79% for experiencing CD4 decline following treatment, however this effect was not statistically significant (RR = 1.79; 95% CI 0.89–3.58). Men were 2 times more likely to experience poor immunological response compared to the women and this difference was statistically significant RR = 2.02, 95% CI 1.23, 3.33). Patients who had been on ART for longer (i.e. 6 months or longer) were less likely to have poor immunological outcomes compared to those who had been on treatment for a shorter duration.

**Table 4:** Table showing crude and adjusted risk ratios for the predictors of immunological response using Poisson regression analysis.

Variable	Categories	Crude Risk Ratios	p value	Adjusted Risk Ratios^+^ (95%CI)	p value
**Age Categories**	Young Adults (18–34yrs)	1.0		1.0	
	Middle aged (35–49yrs)	0.91* (0.88–0.95)		0.86 (0.5–1.47)	0.10
	Older Patients (≥ 50 yrs)	0.92* (0.85–0.99)	0.003	1.79 (0.89–3.58)	
**Gender**	Female	1.0		1.0	
	Male	1.87* (1.35–3.03)	0.028	2.02* (1.23–3.33)	0.005
**Duration on ART**	Less than 6 Months	1.0		1.0	
	6 to 12 Months	0.92 (0.82–1.04)		0.44* (0.21–0.95)	
	13 to 36 Months	0.44* (0.40–0.49)	<0.001	0.36* (0.16–0.56)	<0.001
	Over 36 Months	0.23* (0.19–0.24)		0.13* (0.06–0.27)	

* Significant at 0.05 level, Analysis adjusted for gender and duration on ART

## Discussion

Our study based at a large urban clinic has shown that older age is associated with poorer response to ART and excess mortality compared to the younger. The data agree with other studies across the African continent also showing higher risk for mortality among older HIV infected adults [20, 22–25]. Also in agreement with a recent study from China [26], older persons in our cohort had higher risk for poor immunological outcomes compared to the younger ones. However, this effect was attenuated when we adjusted for duration of ART.

One of the major strengths of our study is that our cohort had been followed for up to 6 years by the time of this analysis. The long duration of follow up in the cohort offers a unique opportunity to examine whether the difference in mortality changes over time. Our data show that older persons retain a significantly higher risk for mortality and this is not modified over time. Some of the African studies have had fairly shorter follow up times, hence not able to make conclusions on the long term survival by age at ART initiation [23].

The higher risk for mortality should be a cause for concern as some countries have shown increasing trends in HIV prevalence among older persons [1] and generally, prevalence in this older group has been shown to be high in many sub Saharan Africa countries, up to 20% in Zimbabwe [27]. The biological explanation for excess mortality may be because of the poor immunological response which is due to involution of the thymus gland among older patients. The involution of the thymus gland, which is involved in CD4 T-cell reconstitution during ART, has a negative effect on immunologic recovery during therapy [28]. Output from the thymus gland reaches its minimum after 55 years of age [29], hence older patients may require adjuvant therapy to stimulate their immunological response to equal that of the younger patients.

Our findings have important implications for treatment for HIV infections among older persons. Current ART treatment guidelines recommend treatment of all children and adolescents who test positive for HIV regardless of CD4 count. Treatment programs have now moved to implement test and treat approach for ART programs. Older persons should be priority for eligibility to initiate ART very early to mitigate the potential poor outcomes in situations where resources are limited. The treatment for older adults is likely to face challenges. First, there is limited data on HIV incidence and prevalence among older persons [30]. Second, older patients are less likely to be screened for HIV [31] as they are considered not sexually active and at lower risk for HIV and therefore are likely to be diagnosed in advanced HIV and present late for treatment. Data from Uganda among a cohort of 750 patients aged 50 and above showed at least 40% of them were sexually active and also older patients in the cohort were more than two times likely to have a Sexually transmitted infection compared to the younger ones [32]. Third, some antiretroviral medicines in the standard regimens are linked to non-communicable diseases or NCDs [33] at the same time patients living to the age when they would be at high risk for NCDs regardless of HIV status. Fourthly, is that older patients have been shown to suffer high levels of stigma, even after disclosure of serostatus [16]. Stigma among older persons has also been shown to have a positive correlation with depression [15, 34], and all of these will present challenges of successful ART delivery to these patients.

This study also showed a steady rise in the proportion of young adults initiated on ART from 43.7% in 2006 to 63.7% in 2012 with a decrease in the proportion of patients in the mid-age category. These findings may mean two things; firstly it could be that young adults have continued to actively access HIV care and treatment more than the middle aged and the older patients. Secondly this data suggests that younger people may be more likely to acquire HIV compared to the older patients but we should note that this prevalence data and not incidence and hence cannot be used to measure new infections. However, the number of new patients initiated on ART could be used as a proxy assuming there are no barriers to accessing ART among the different age groups. As a result, our data suggests that HIV infection prevention packages should target the young adults during comprehensive HIV/AIDS services delivery since they formed the majority of new patients initiated on ART and possibly new HIV infections.

The proportion of older adults enrolling into ART programs remained stable for most of the follow up and showed modest decreases towards the end of the follow up period. Our data are different than those of a recent publication from a large cohort in South Africa where the proportion of patients aged 50 years and above increased from 6% to 10% over a ten year period [22], indicating that this program has been successful in reaching older clients or that prevalence of HIV is increasing in this age group. An almost flat line in the trend of enrollment in our cohort may imply that older persons are less likely to be diagnosed, have poor access to ART because of certain barriers or older persons represent a lower risk group or a combination of these factors. There is limited data on the prevalence of HIV among older persons in Africa but available data from the few countries that have this data such as South Africa indicate a high prevalence and incidence in this age group [35].

CD4 count is an important confounder in our study. Our data also show significant trends in CD4 count. Patients initiating ART in 2012 were more likely to start ART at higher CD4 count than those who initiated in 2006 and this may be explained by the policy change in the guidelines for ART initiation in 2010 when the CD4 cut off for ART initiation was lifted from 250cells/ml to 350cells/ml. However, data show the increase in the percentage of patients initiating ART at higher CD4 counts started before the policy change. This rise may be explained by more availability of ART treatment slots at this clinic as more resources for treatment became available. The increasing CD4 counts were independent of age. Despite this, our results indicated higher risk of mortality among older patients and remained significant even after adjusting for CD4 count.

Overall, our data show the largest proportion of patients receiving care at this large urban clinic is predominantly young and female. Over 60% of the patients were female with an overall median age of 32 years. The distribution is similar to that seen at several other clinics in sub Saharan Africa [36, 37], suggesting a feminization of the HIV epidemic on the continent.

Our study has several weaknesses. First, we used routinely collected data from a large busy clinic and such data often has several missing values. Clinicians may miss to complete some fields because of the rush to complete the long queues of patients for consultation visits. For instance, over 100 patients had no ART start date and over 600 transferred out and there was no medical record for them. If patients transferred out are more likely to be older, this would result in an underestimate of total mortality. However, the gender and age distribution of patients not included in the analysis were similar to those in the analysis. Second, mortality may also have been under-estimated in cases where patients have died and mortality is not captured in the database, and instead clinicians may capture this as a loss to follow up. Third, we did not have viral load results for the entire cohort. VL testing is a gold standard for monitoring ART but is expensive and patients have to cost share to get the test done. Fourth, we collected these data six years ago and policies in HIV treatment initiation have changed. However, the data are still relevant because at least 40% of the population have still not tested for HIV. Additionally, because of absence of these data, we did not adjust for chronic diseases such as hypertension and diabetes which are major confounders in this study. Lastly, we did not include adherence in the analysis because these data were not routinely collected for majority of patients. However, literature shows older patients adhere better to their ART medications [38], hence adjusting for adherence would not dampen the measure of effect.

In conclusion, our study has shown that the proportion of young adults and those patients in WHO Stage I of HIV disease who were being initiated on ART increased over a seven year period. Secondly, older patients showed poorer immunological response and higher risk for mortality following ART initiation compared to the young adults. Further studies should be conducted to further understand the biological mechanisms for the poorer immunological response and the excess mortality in the older patients. Interventions such as adjuvant therapy to antiretroviral therapy may be necessary to improve immunological recovery.

## Supporting information

S1 Data Age_data—Data set for cohort of patients receiving antiretroviral therapy. (DTA)
